# Predicting complicated appendicitis is possible without the use of sectional imaging—presenting the NoCtApp score

**DOI:** 10.1007/s00384-023-04501-x

**Published:** 2023-08-19

**Authors:** Jens Strohäker, Martin Brüschke, You-Shan Feng, Christian Beltzer, Alfred Königsrainer, Ruth Ladurner

**Affiliations:** 1 Department of General, Visceral and Transplantation Surgery, University Hospital of Tuebingen, Hoppe-Seyler-Straße 3, 72076 Tuebingen, Germany; 2 Department of Epidemiology and Biostatistics, University Hospital of Tuebingen, Tuebingen, Germany; 3 Department of Surgery, Armed Forces Hospital, Ulm, Germany

**Keywords:** Complicated appendicitis, Logistic regression, Area under the curve, Prediction models

## Abstract

**Purpose:**

Appendicitis is among the most common acute conditions treated by general surgery. While uncomplicated appendicitis (UA) can be treated delayed or even non-operatively, complicated appendicitis (CA) is a serious condition with possible long-term morbidity that should be managed with urgent appendectomy. Distinguishing both conditions is usually done with computed tomography. The goal of this study was to develop a model to reliably predict CA with widespread available clinical and laboratory parameters and without the use of sectional imaging.

**Methods:**

Data from 1132 consecutive patients treated for appendicitis between 2014 and 2021 at a tertiary care hospital were used for analyses. Based on year of treatment, the data was divided into training (*n* = 696) and validation (*n* = 436) samples. Using the development sample, candidate predictors for CA—patient age, gender, body mass index (BMI), American Society of Anesthesiologist (ASA) score, duration of symptoms, white blood count (WBC), total bilirubin and C-reactive protein (CRP) on admission and free fluid on ultrasound—were first investigated using univariate logistic regression models and then included in a multivariate model. The final development model was tested on the validation sample.

**Results:**

In the univariate analysis age, BMI, ASA score, symptom duration, WBC, bilirubin, CRP, and free fluid each were statistically significant predictors of CA (each *p* < 0.001) while gender was not (*p* = 0.199). In the multivariate analysis BMI and bilirubin were not predictive and therefore not included in the final development model which was built from 696 patients. The final development model was significant (x^2^ = 304.075, *p* < 0.001) with a sensitivity of 61.7% and a specificity of 92.1%. The positive predictive value (PPV) was 80.4% with a negative predictive value (NPV) of 82.0%. The receiver operator characteristic of the final model had an area under the curve of 0.861 (95% confidence interval 0.830–0.891, *p* < 0.001. We simplified this model to create the NoCtApp score. Patients with a point value of ≤ 2 had a NPV 95.8% for correctly ruling out CA.

**Conclusions:**

Correctly identifying CA is helpful for optimizing patient treatment when they are diagnosed with appendicitis. Our logistic regression model can aid in correctly distinguishing UA and CA even without utilizing computed tomography.

## Introduction

Acute appendicitis is among the most common diagnoses in emergency rooms and general surgery departments with an annual incidence of nearly 100 cases per 100,000 adults [1]. While it is considered a disease of the younger population, it is also frequently encountered in the elderly [2]. Prediction models for appendicitis have been reported by several groups. The most well known and most commonly used risks scores are the Alvarado score and the Appendicitis Inflammatory Response (AIR) score [3, 4] which use clinical and laboratory data to predict appendicitis. The seminal APPAC I and APPAC II trial have shown favorable outcomes after non-operative management in the absence of complicated appendicitis (CA) [5, 6], which has become an alternative to surgery in current clinical practice for uncomplicated appendicitis (UA) [5, 7, 8]. Before inclusion into the trial, CA (perforation, gangrene, or abscess) had to be ruled out via computed tomography (CT) [5, 6], which harbors an inherent risk of cancerogenicity due to ionizing radiation, especially in the young [9]. In countries that are prone to medical litigation suits, nearly all patients undergo CT scans during the diagnostic process. The MUSTANG Trial reported that 90% of patients in the USA that are diagnosed with appendicitis had preoperative CT scans, and a CT scan is considered the appropriate tool to diagnose appendicitis by the American College of Radiology [10, 11]. In Europe, the imaging of choice is a focused ultrasound [12–14]. However, there is limited data on the discrimination of UA and CA without the use of a CT or magnetic resonance imaging (MRI) [15].

The primary goal of this study was to develop a prediction model for complicated appendicitis in a patient cohort that underwent appendectomy at a tertiary teaching facility without the use of sectional imaging and to compare its predictive potential to the Atema score and C-reactive protein (CRP) alone. The secondary goal was to develop a predictive risk score for easy clinical application.

## Materials and methods

### Study cohort

**Fig. 1:**
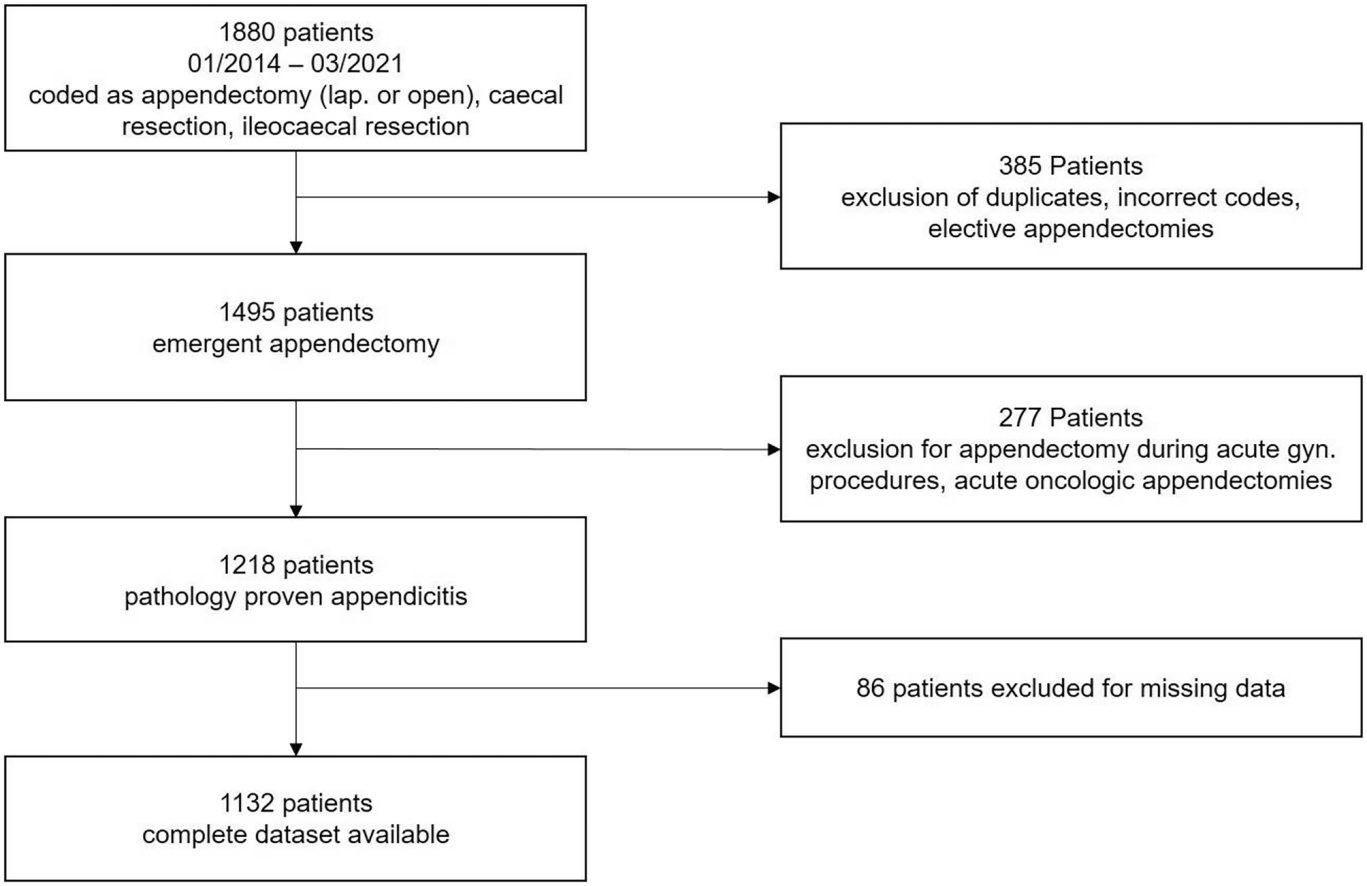
Study cohort flow diagram

We retrospectively analyzed a convenient cohort of 1880 consecutive adult patients (age ≥ 16 years) that underwent appendectomy at our tertiary teaching facility between 2014 and 2021. Patients were identified with the procedure codes for appendectomy, partial cecectomy or ileocecal resection. After exclusion of duplicates, the operation reports were screened by a clinician to exclude patients with incorrect procedure codes or indications other than acute appendicitis. Patients that had elective appendectomy or appendectomy during other procedures were excluded from the analysis, as were all patients who were taken to the operating room for the suspicion of appendicitis but were ultimately diagnosed to have different diseases (mainly gynecological infections or inflammatory bowel diseases). Ultimately 1218 patients fulfilled the criteria of having been treated surgically for acute appendicitis. For details, see Fig. 1. None of these patients was treated antibiotically. All patients underwent surgical resection.

Given the size of the dataset (with more than 400 cases of complicated appendicitis), no formal sample-size calculation was performed. However, according to a previous simulation study sample sizes of > 500 with 50 events per predictor are considered adequate and reasonably close to population level patterns [16].

### Clinical definitions

CA was defined as the presence of gangrenous or necrotizing appendicitis, local abscess, perforation of the appendix or the presence of peritonitis. All other patients were classified as having UA. All diagnoses were clinically made by the surgeon and histopathology did not alter this classification since the purpose was to determine postoperative care, which should not be based on pathological results available 48 h after the procedure.

### Model building

Only patients with complete data were included in analyses: *n* = 1132. The majority of missing data were due to lacking preoperative ultrasound (in favor of primary computed tomography). For the univariate analysis, we assessed the following parameters: age, gender, body mass index (BMI), American Society of Anesthesiologists (ASA) score, duration of symptoms in full days (rounded appropriately) at the time of presentation to the emergency department, free fluid on ultrasound, white blood count (WBC), CRP, and total bilirubin.

The following parameters were not assessed: body temperature at presentation, diameter of appendix on ultrasound, presence of appendicoliths on ultrasound (all three due to inconsistent documentation in the admission charts or ultrasound reports), procalcitonin, delta neutrophil index, neutrophil-to-lymphocyte ratio (all three not routinely ordered during preoperative labs).

To allow for validation the patients were split up into two groups. The development cohort included 696 patients (61.5%) that were treated before 2019. The validation cohort was made up of the 436 patients (35.5%) treated from 2019.

### Simplification for clinical use

In order to facilitate clinical use of our prediction model, we simplified the outcome parameters. In order to identify appropriate cutoff values, we classed the linear parameters into ordinal categories that then were collapsed if they had similar odds when compared to the reference. This process was repeated until there were no more than three outcomes for age, WBC, and CRP. The threshold values were defined to be comparable to the Atema score.

Age was grouped as Age0, ≤ 40 years; Age1, 40.01 to 60 years; Age2, > 60 years.

ASA was grouped as ASA0 ≤ ASA II; ASA1 was ASA III.

Gender was grouped as Gender0 = female; Gender1 = male.

Symptom duration was grouped as Symptoms0, ≤ 2 days; Symptoms1, > 2 days.

WBC was grouped as WBC0 ≤ 14,000 white cells; WBC1 = 14,001 to 18,000 white cells; WBC 2 > 18,000 WBC.

CRP was grouped as CRP0 ≤ 5 mg/dl; CRP1 = 5.01 mg/dl to 10 mg/dl, CRP2 > 10 mg/dl.

Free fluid on ultrasound was grouped as FF0 = no free fluid on US; FF1 free fluid on US.

### Reference standard test

There is no reference standard test to diagnose complicated appendicitis. It is usually diagnosed clinically during surgery. Likely to most commonly accepted reference standard test pre-op would be a CT scan. Given the nature of our clinical pathways and the low number of CT scans performed, a comparison with CT results was not possible for the lack of available data. The lack of data availability was also the reason the Atema score was not used as the reference standard. Therefore, we decided to define CRP in mg/dl as our reference standard, for its completeness of data and widespread clinical use. Both the outcomes of our model and the prediction of CA with CRP had dichotomous outcomes, for which there were no indeterminate test results.

### Transparent reporting

Our aim was to report our results according to the Standards for Reporting Diagnostic Accuracy (STARD 2015) guidelines [17].

### Statistical analyses

Comparison between the patients with complicated and uncomplicated appendicitis was carried out using chi-square (Χ^2^) test or Fisher’s exact test for nominal variables and Mann-Whitney *U*-test (MWU) for continuous variables, as appropriate. Comparisons with *p* values less than 0.05 were considered to be statistically significant. All *p* values in this manuscript are results of two-sided testing. Bonferroni-correction was applied where necessary. Binary logistic regression was used to build prediction models: odds ratio (OR) with 95% confidence intervals (95% CI) are presented in this paper. First, all individual predictors were assessed in univariate models. These parameters were then combined in a multivariate model. For the model building, Harrell’s rule of ten was applied [18]. We calculated the sensitivity, specificity as well as positive and negative predictive values for the model predictions. To calculate diagnostic accuracy of our model, area under the receiver operator characteristics curve (AUROC) was assessed for candidate models).

All statistical analyses were carried out using IBM SPSS Statistics for Windows, Version 28.0 (IBM Corp., Armonk, NY, USA).

## Results

### Patient characteristics

The final patient cohort of 1132 patients with no missing data is summarized in Table 1. The table also shows the patient characteristics of included patients across appendicitis subgroups. Of the 1132 patients, 729 were diagnosed with UA and 403 with CA. The gender distribution was similar for both groups. Patients in the CA group were significantly older (48.2 vs. 32.1 years, MWU < 0.001) and reported a longer duration of symptoms (median of 2 vs. 1 days, MWU < 0.001). The ASA score was generally higher in the complicated group (Χ^2^ *p* < 0.001) and free fluid on preoperative ultrasound was more common in the complicated group (Χ^2^ *p* < 0.001).

**Table 1:** Patient cohort and comparison of the uncomplicated and complicated appendicitis

	Patient cohort (*n* = 1132)	Uncomplicated appendicitis (*n* = 729)	Complicated appendicitis (*n* = 403)	*P* value
	Mean (SD) or *n* (percentage)			
Age	37.9 (± 17.4)	32.1 (± 13.9)	48.2 (± 18.4)	< 0.001^a^
Sex, male	603 (53.3%)	378 (51.9%)	225 (55.9%)	0.199^b^
BMI, kg/m^2^	25.5 (± 5.1)	25.0 (± 5.0)	26.4 (± 5.2)	< 0.0010^a^
ASA score - ASA I - ASA II - ASA III	615 (54.3%) 452 (39.9%) 65 (5.7%)	465 (63.8%) 250 (34.3%) 14 (1.9%)	150 (37.2%) 202 (50.1%) 51 (12.7%)	< 0.001^b^
Duration of symptoms in days, median (IQR)	1 (Q25 1; Q75 2; IQR 1)	1 (Q25 1; Q75 2; IQR 1)	2 (Q25 1; Q75 3; IQR 2)	< 0.001^a^
WBC × 10^3^/µl	13.4 (± 4.8)	12.7 (± 4.2)	14.6 (± 5.6)	< 0.001^a^
C-reactive protein, mg/dl	5.8 (± 6.8)	3.2 (± 3.8)	10.4 (± 8.4)	< 0.001^a^
Total bilirubin, mg/dl	1.1 (± 0.8)	1.0 (± 0.7)	1.2 (± 0.8)	< 0.001^a^
Free fluid on ultrasound	489 (43.2%)	268 (36.8%)	221 (54.8%)	< 0.001^b^

Table 1 shows the univariate comparison of the uncomplicated and complicated appendicitis population

*SD* standard deviation, *BMI* body mass index, *ASA* American Society of Anesthesiologists, *IQR* interquartile range, *Q25* 25^th^ percentile, *Q75* 75^th^ percentile, *WBC* white blood count

^a^Mann-Whitney *U*-test

^b^Χ^2^ test

### Model building

In order to identify predictors, univariate binominal logistic regression was performed for candidate predictor measures. The odds ratio and 95% CI for each predictor variable are displayed in Table 2. With regard to previously published studies, the following parameters were evaluated: age, gender, BMI, ASA score, free fluid on ultrasound, WBC, CRP, and bilirubin. WBC was rounded to the next 1000 and CRP to a single digit without decimals. Except for gender all parameters appeared to be potential predictors of CA.

**Table 2:** Univariate predictors of complicated appendicitis

	Odds ratio	95% CI	*P* value
Age in years	1.060	1.051–1.069	< 0.001
Sex, male	1.174	0.919–1.499	0.199
BMI in kg/m^2^	1.054	1.029–1.080	< 0.001
ASA score - ASA 1 - ASA 2 - ASA 3	1.000 2.505 11.293	Reference 1.929–3.253 6.079–20.979	< 0.001 < 0.001
Duration of symptoms in days	1.053	1.013–1.096	0.010
WBC × 10^3^/µl	1.096	1.065–1.128	< 0.001
C-reactive protein in mg/dl	1.231	1.196–1.267	< 0.001
Total bilirubin in mg/dl	1.463	1.224–1.750	< 0.001
Free fluid on ultrasound	2.089	1.631–2.675	< 0.001

Table 2 shows the univariate prediction models

*CI* confidence interval, *BMI* body mass index, *ASA* American Society of Anesthesiologists, *IQR* interquartile range, *Q25* 25^th^ percentile, *Q75* 75^th^ percentile, *WBC* white blood count

### Correlation of linear parameters

In order to rule out collinearity of the parameters of the final model cohort, we performed Pearson’s correlation for age, duration of symptoms WBC, and CRP both for the overall cohort, the uncomplicated and the complicated subgroups. In the overall cohort except for the age and CRP none of the correlations was more than weakly correlated (age and CRP *ρ* = 0.345, *p* < 0.001). For details, see Table 3.

**Table 3:** Pearson’s correlation to rule out collinearity

	Age	Duration	WBC	CRP
Age				
Duration of symptoms	0.059*			
WBC	0.027	−0.091**		
CRP	0.345***	0.061*	0.154***	

Table 3 shows the Pearson’s correlation coefficients for the linear parameters in the final regression model. While there are significant correlations amongst these, none is strongly correlated according to Cohen’s correlation effect size (i.e., *ρ* > 0.5)

*WBC* white blood count, *CRP* C-reactive protein

*p < 0.05; **p < 0.01; ***p < 0.001

### Multivariate analysis of a development model and validation

**Fig. 2:**
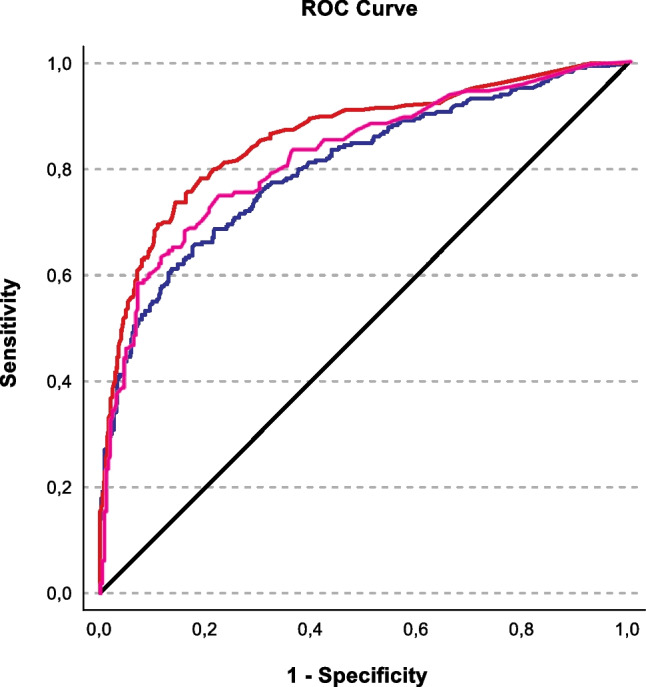
Receiver operator characteristic (ROC) analysis of the development model (red). Area under the curve 0.861 (95% CI 0.830–0.891) compared to the validation model (pink) 0.823 (95% CI 0.781–0.865) and CRP (blue) 0.804 (95% CI 0.768–0.840)

To allow for validation the sample was split into a development dataset (patients treated before 2019, *n* = 696, 61.5%) and a validation dataset (patients treated 2019 or after, *n* = 436, 38.5%). The predictors were then combined into the development model. Both BMI and total bilirubin, while being univariate predictors of CA, were removed from the model since they were not statistically significant predictors and did not substantively improve overall model fit. The development model was significant (x^2^ = 304.075, *p* < 0.001) which indicates the capability of the model to distinguish uncomplicated from complicated appendicitis. The model accounted for approximately 35.4% (Cox and Snell *R*^2^) to 48.9% (Nagelkerke’s *R*^2^) which indicates a moderate to strong model and was able to correctly identify 81.6% of disease severity. The Hosmer-Lemeshow goodness of fit test was not significant (p = 0.636) indicating that the model has a sufficient fit. The sensitivity of this model was 61.7% with a specificity of 92.1%. The positive predictive value (PPV) was 80.4% with a negative predictive value (NPV) of 82.0%. The receiver operator characteristic (ROC) analysis of the development model (Fig. 2) had an area under the curve (AUC) of 0.861 (95% CI 0.830–0.891, *p* < 0.001).

**Table 4:** Multivariate analysis of predictors in the development cohort, *n* = 696

	B	S.E	Wald	Odds ratio	95% CI	*P* value	Points
Constant	−3.151	0.275	130.955	0.043			
Age							
- ≤ 40			55.205			< 0.001	0
- 40–60	1.374	0.242	32.224	3.951	2.459–6.350	< 0.001	4
- > 60	1.952	0.314	38.783	7.046	3.81–13.027	< 0.001	6
Sex, male	0.313	0.210	2.220	1.367	0.906–2.062	0.136	1
ASA score 3	1.070	0.551	3.766	2.916	0.989–8.594	0.052	3
Symptoms > 2 days	0.766	0.154	9.103	2.150	1.308–3.535	0.003	2
WBC							
- ≤ 14.000			24.601			< 0.001	0
- 14–18 × 10^3^	0.684	0.259	8.174	1.981	1.240–3.165	0.004	2
- > 18000	1.438	0.300	23.042	4.213	2.342–7.580	< 0.001	4
CRP							
- ≤ 5 mg/dl			78.163			< 0.001	0
- 5.01–10 mg/dl	0.992	0.259	14.652	2.697	1.623–4.482	< 0.001	3
- > 10 mg/dl	2.385	0.273	76.095	10.864	6.357–18.568	< 0.001	7
Free fluid (US)	0.625	0.210	8.869	1.868	1.238–2.818	< 0.003	2

Table 4 shows the binary logistic regression model calculated from the simplified parameters

*SE* standard error, *CI* confidence interval, *ASA* American Society of Anesthesiologists, *WBC* white blood count, *CRP* C-reactive protein, *US* ultrasound

Using the scoring wizard function of SPSS, the developed model was then applied to the remaining data in the validation dataset. Of the patients in the validation cohort, 96 were correctly identified as complicated appendicitis out of 119, whereas 250 were correctly identified as uncomplicated out of 317. The sensitivity was 56,9% with a specificity of 91.6%. The NPV was 78.9% with a PPV of 80.7% and an accuracy of 79.4%. The AUROC was 0.823 (95% CI 0.781–0.865, *p* < 0.001).

**Fig. 3:**
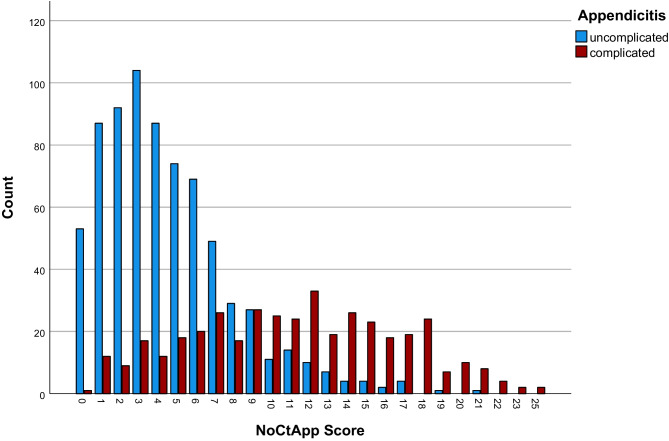
Distribution of total risk scores in the uncomplicated and complicated group

The Beta regression coefficients were multiplied by three and rounded to the next whole number to create a scoring system from which the NoCtApp score was calculated by addition of the seven individual point values each patient was awarded. The distribution is shown in Fig. 3. The minimum number of points was 0 whereas the maximum NoCtApp score was 25. The distribution of scores between the CA and UA patients is depicted in Fig. 3. In order to avoid underdiagnosing complicated appendicitis the goal was to define an acceptable false-negative rate of around 5%. Twenty-two patients with CA had a NoCtApp score ≤ 2 accounting for 5.5% of CA. A NoCtApp score ≤ 2 has a sensitivity of 94.5% with a specificity of 68.2%. The NPV was 95.8% with a PPV of 62.2% and an accuracy of 77.8%.

### Comparison of the model to the reference standard CRP

**Fig. 4:**
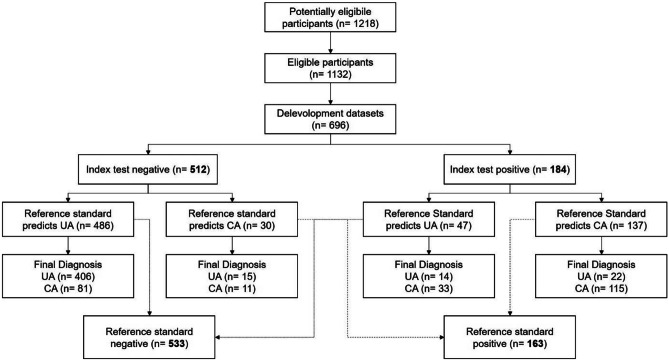
Flow diagram according to STARD 2015

Our development model predicted 148/184 (PPV = 80.4%) cases of CA as well as 420/512 (NPV = 82.0%) of UA correctly. It had diagnostic accuracy of 81.6% with a sensitivity of 61.7%, a specificity of 92.1%. The AUROC for the model was 0.861 (0.830–0.891) compared to CRP (blue) 0.804 (0.768–0.840). CRP by itself predicted 126/163 (PPV = 77.3%) cases of CA and 419/533 (NPV 78.6%) of UA correctly. It had an accuracy of 78.3% with a sensitivity of 52.5% and a specificity of 91.9%. For details, see Fig. 4.

## Discussion

As a result of this study, we propose the NoCtApp score to be used as a clinical risk score for the differentiation of uncomplicated and complicated appendicitis based on easily reproducible parameters (age, gender, ASA score, symptom duration, free fluid on US, WBC, and CRP) that can be calculated within seconds. While treatment for UA was both shown to be safely delayed or even non-surgical, misdiagnosing CA can have severe consequences for the patient. While our score ranges from 0 to 25 points we set the cutoff at ≤ 2 vs > 2 points, since this has a NPV of 95,8%. While around 95% of patients with CA had a NoCtApp of > 2, so did more than half of UA patients. In order to avoid falls negatives. Patients with a NoCtApp score of > 2 points should be offered early laparoscopy or sectional imaging.

Over the last 40 years, many studies have focused on the value of clinical, laboratory and imaging parameters in the diagnosis of appendicitis. Individual parameters were combined to several risk scores of which the most widely accepted ones are the Alvarado and the AIR score [3, 4]. In 2021, Andersson et al. published prospective follow-up data on 3878 patients assessed with their AIR score. They proposed outpatient follow-up for low risk scores, surgical exploration for high risk scores and either sectional imaging or short-term clinical reassessment or extended imaging for medium risk scores.

CT scans for the diagnosis of appendicitis are the diagnostic standard in the US and most studies rely heavily on sectional imaging [19–22]. Sectional imaging is less frequently utilized in Europe due to its cost, harm from radiation and the widespread availability of focused ultrasound [13, 23]. Additionally, CTs have been reported to underdiagnose CA. In up to 22% of patients were found to have complicated disease during surgery [24]. Bolmers et al. recently reported a diagnostic accuracy of CTs for the distinction of CA from UA with a sensitivity of 45%, a specificity of 88% and a NPV of 58% [14]. A recent multicentric British study by Javanmard-Emamghissi et al. demonstrated favorable outcome for antibiotically managed appendicitis. They reported a 70% rate of CT scans in some 3400 patients. While this is comparably low to other randomized trials, they do not report on the outcomes of the patients that did not undergo a CT or how they concluded that the remaining 30% could be managed without a CT-scan [25]. It goes without saying, that while we generally strive to avoid iodizing radiation, CTs can detect unexpected differentials and cases of appendicitis secondary to (obstructing) tumors [26, 27].

There is no formal gold standard test that could serve as a reference standard for our model/ index test. Given our low rate of CT scans we are unable to compare our test results to CT results. Arguably the most similar reference would be the 2015 Atema score. Atema et al. published a scoring system to distinguish UA from CA in 2015 in 395 patients that were taken to the OR for an appendectomy. They proposed two models on the base of clinical, laboratory and imaging findings (comparing computed tomography to ultrasound). Their ultrasound model was created from 312 patients and had an AUC of 0.82 for correctly predicting CA [28]. The most obvious reference standard for our study would be the Atema score from 2015, unfortunately not all the predictors presented in their study were available in our dataset (or clinical database) [28]. In our literature research, it was the most widely accepted risk score that did not make use of sectional imaging. Similar to the Atema score for ultrasound-driven decision-making, we explored our data and developed a model from 696 patients operated before 2019 that we then validated with the 436 patients that were treated from 2019 onwards. Their final model included age ≥ 45 years, white cell count > 13,000/ml, CRP in mg/dl ≤ 5, 5.1–10 or >10, duration of symptoms ≥ 2 days and free fluid on ultrasound in their model. Additionally, that model contained core body temperature in °C ≤ 37.0, 37.1 – 37.9 or ≥ 38.0 and ultrasound finding of an appendicolith. The same group performed a retrospective follow-up study to evaluate their model’s negative predictive value to rule out CA to serve as a selection tool for studies. That model correctly identified 93.8% of the patients that presented with UA [29]. Our model included different cut-offs for age and white cell counts, as well as gender and ASA score but not body temperature and appendicolith and is therefore likely easier to reproduce and less likely biased from errors in temperature measurement or ultrasound interobserver disparities. Our model had an AUROC of 0.861 in the score development sample and performed well in the validation cohort with an AUROC of 0.823. The model was then modified into the NoCtApp risk score that can be easily adapted to the clinical setting in the for selecting patients to consider for sectional imaging or prioritize in the operating room schedule.

Several individual laboratory parameters have been discussed as independent predictors of CA. The most commonly accepted one is elevated C-reactive protein (Xu et al. > 3.8 mg/dl, Moon et al. > 7.05 mg/dl, Akai et al. > 0.5 mg/dl OR 10.15), which was reported to have an AUC of 0.796 – 0.853 to detect CA [21, 30–32]. This matched our results in that we found CRP to be the strongest individual predictor of CA. Therefore, we decided to define CRP as the reference standard for our model. CRP alone was able to correctly predict 78.3% of the cases correctly and had with an AUC of 0.804 similar discriminatory power as described in the aforementioned studies.

Other parameters are discussed more controversially, such as bilirubin. While some found hyperbilirubinemia to be predictive of appendicitis or more specifically perforation, others were less keen about the predictive benefits [33–36]. While we did find hyperbilirubinemia to be more common among patients with CA in univariate analysis, it did not add predictive accuracy to the multivariate model and was not retained. Less widely accepted parameters include hyponatremia [37] and hyperfibrinogenemia [38, 39], which we did not routinely study and therefore could not include in our model.

When in doubt surgical exploration or a CT scan can and should be discussed with the patient for patient- “assisted” decision-making process. Our general practice is to avoid CT scans in patients under 40 years and to rather take them to OR for laparoscopy since they are at an increased risk of developing radiation-induced malignancy in the future [9]. Our risk score aids in identifying CA correctly and prioritize these patients on the theater schedule.

## Strength and limitations

With all diagnostic regression modelling studies, there are limitations to be discussed. All data included in this study were collected at the same institution. We used a convenient dataset for our analysis for which we did not perform case sample size calculation or did prospective data collection. While this is a sizeable cohort with 696 patients for the model development and 436 patients to validate the model in, not being able to control which parameters to collect made comparison to a reference standard nearly impossible. This is further complicated by the fact that no gold standard has been defined/accepted for the diagnosis of CA. This poses a methodical challenge to the researcher, given that there is no obvious reference standard to compare one’s model to. Just by the nature of our clinical care we were unable to use CT results or Atema score as the reference. Given the retrospective analysis, the diagnosis of CA was made based on information taken from the operation reports. There was a risk for bias given that there were no routine intraoperative images available for secondary assessment by the study team. Lastly, this model was created with a dataset of proven cases of acute appendicitis. A universal clinical application of such a model is limited by the complex differential diagnoses to appendicitis which need to be considered.

## Conclusion

Predicting acute complicated appendicitis without sectional imaging with the aid of our regression model appears to be possible. Its’ predictive value was higher than that of CRP and similar to the Atema score but potentially easier to reproduce. While the NoCtApp score predicts complicated appendicitis fairly well, the model should be tested in a prospective cohort potentially with external validation before clinical implementation.

## Data Availability

Anonymized data can be provided on further request.
